# Tumeur brune du maxillaire révélatrice d'hyperparathyroidie primaire: à propos d'un cas et revue de la littérature

**DOI:** 10.11604/pamj.2013.14.21.1359

**Published:** 2013-01-15

**Authors:** Fassih Malika, Loubna Taali, Mohamed Akssim, Abada Reda, Sami Rouadi, Mohamed Mahtar, Mohamed Roubal, Mustapha Essaadi, Mohamed Fatmi El Kadiri

**Affiliations:** 1 Service d'ORL et chirurgie Cervico-faciale, hôpital 20 Aout, CHU Ibn Rochd, Casablanca, Maroc; 2 Laboratoire d'anatompapathologie, hôpital Ibn Rochd, Casablanca

**Keywords:** Tumeur brune, hyperparathyroïdie, tumeur à cellules géantes, maxillaire, adénome parathyroidien, brown tumor, Hyperparathyroidism, giant cell tumor, maxillary, Parathyroid adenoma

## Abstract

Les tumeurs brunes sont des lésions ostéolytiques rarement révélatrices des hyperparathyroïdies. Elles surviennent habituellement au stade terminal de l'hyperparathyroïdie primaire ou secondaire. Durant les 3 dernières décennies, le diagnostic des hyperparathyroïdies est le plus souvent fait à la phase asymptomatique grâce aux dosages systématiques du calcium et l'avènement des nouvelles techniques, de dosage de la parathormone. Nous rapportons l'observation d'une patiente avec hyperparathyroïdie primaire révélée par une tumeur du maxillaire, dont la TDM avait mis en évidence un processus ostéolytique agressif. L'intervention chirurgicale a consisté en une maxillectomie droite avec reconstruction. Le résultat anatomo-pathologique a conclu en une tumeur à cellule géantes bénigne du maxillaire. Le diagnostic de tumeur brune a été évoqué et confirmé après la réalisation d'un bilan phosphocalcique qui a mis en évidence une hypercalcémie, avec une hypophosphorémie. La recherche étiologique a objectivé à la TDM cervicale un processus en situation rétro et infra thyroïdienne droite en faveur d'un adénome parathyroïdien. Le dosage de la parathormone: 322 pmol/L a confirmé le diagnostic. Nous rappelons à travers cette observation la difficulté d’établir un diagnostic correct chez les patients avec un processus ostéolytique du maxillaire et la nécessité de rechercher une hyperparathyroïdie devant une lésion à cellules géantes vue le caractère insidieux de cette endocrinopathie.

## Introduction

La tumeur brune est une entité exceptionnelle dans la pratique d'un ORL et chirurgien maxillo-facial. C'est une lésion non néoplasique résultant d'une anomalie du métabolisme osseux dans le cadre d'une hyperparathyroïdie primaire ou secondaire. Elle est le résultat direct de l'action de la parathormone sur la trame osseuse. Elle est secondaire, dans la majorité des cas, à une hyperparathyroïdie primaire qui résulte dans plus de 80% des cas d'un adénome parathyroïdien [1]. Elle est rapportée chez 4.5% des patients avec une hyperparathyroïdie primaire et 1.5 à 1.7% de ceux avec une hyperparathyroïdie secondaire [2].

En 1934, Albright en a fait la première description au niveau du squelette facial [3]. La tumeur brune peut affecter tout le squelette osseux. L'atteinte maxillo-mandibulaire demeure inhabituelle [4]. En effet, elle se manifeste par une tuméfaction jugale progressive, douloureuse. Elle peut être agressive localement sans potentiel métastatique [5]. Histologiquement, elle fait partie des lésions à cellules géantes des maxillaires [6]. Par conséquent, le diagnostic de certitude requière des investigations systémiques: dosage du calcium et de la PTH afin de différentier entre ces lésions.

## Patient et observation

**Figure 1:**
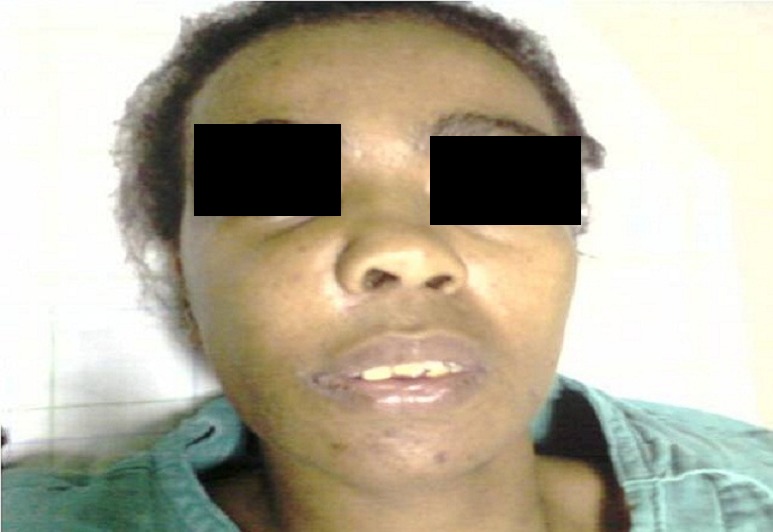
Tumeur maxillaire droite entrainant une déformation et asymétrie de la face avec des pertes et des déplacements dentaires

**Figure 2:**
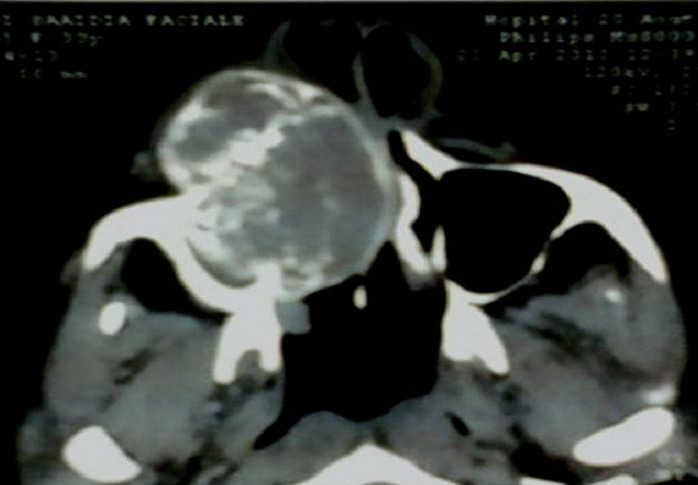
TDM maxillaire en coupe axiale, importante tumeur ostéolytique du maxillaire droit envahissant le sinus maxillaire et la fosse nasale, de densité tissulaire hétérogène, avec des calcifications soufflant et rompant la corticale

**Figure 3:**
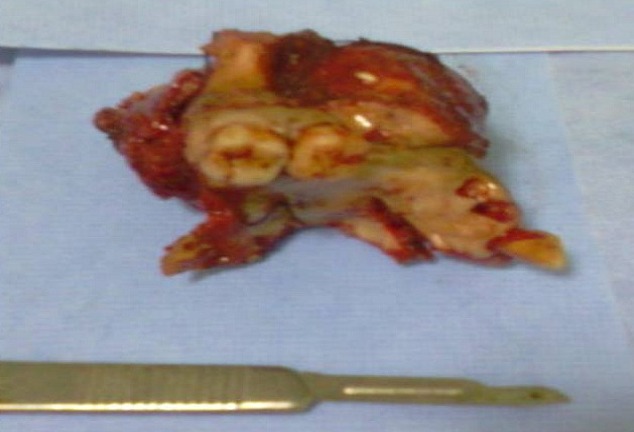
Pièce de la maxillectomie médiane: Aspect de la tumeur après exérèse

Patiente âgée de 32 ans, adressée au service d'ORL et chirurgie cervico-faciale de l'hôpital 20 Aout de Casablanca, pour une tuméfaction jugale droite douloureuse, évoluant progressivement depuis 1 année, avec obstruction nasale, sans autres signes associées, notamment pas d’épistaxis, ni signes oculaires. L'anamnèse ne retrouve aucun antécédent pathologique particulier, notamment pas d'histoire familiale d'hyperparathyroidie, ou autre endocrinopathie. L'examen clinique avait mis en évidence une asymétrie faciale due à une tumeur gingivo-maxillaire dure, comblant le vestibule supérieur et entrainant une voussure du palais. Des déplacements avec mobilité dentaires ont été observés dans la zone atteinte, avec chute de la 12^eme^et 13^eme^dent (Figure 1). Le reste de l'examen somatique n'a pas révélé d'anomalies, notamment pas d'adénopathies ni de douleurs osseuses. La tomodensitométrie a objectivé une masse tissulaire ostéolytique hétérodense, largement nécrosé, siège de calcifications, centré sur le sinus maxillaire droit mesurant 52 mm/35mm, s’étendant en dedans dans les cellules éthmoïdales et la fosse nasale homolatérale refoule et infiltre la cloison nasale. En haut, il lyse le plancher de l'orbite et infiltre la graisse extra conique. En bas, il lyse le palais osseux et bombe dans la cavité buccale et lyse le processus alvéolaire, en avant, il lyse la paroi antérieure du sinus maxillaire et s’étend dans les parties molles en regard (Figure 2, Figure 3). La rhinocavoscopie a révélé une masse comblant totalement la fosse nasale droite sans altération de la muqueuse pituitaire. Une biopsie tumorale a été réalisée dans le même temps, elle a objectivé un aspect en faveur d'un ostéosarcome.

**Figure 4:**
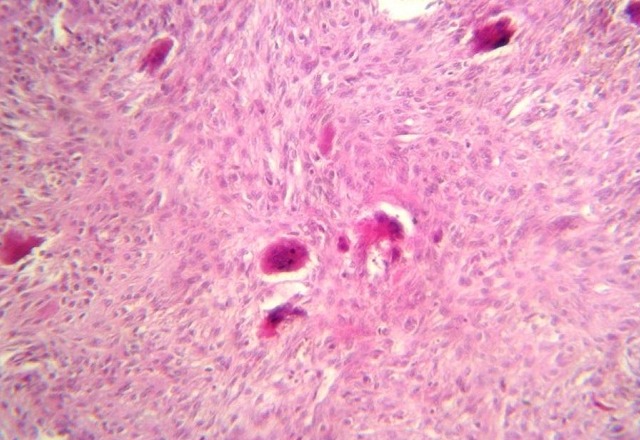
H.E x 200: Prolifération cellulaire faite d'un contingent mononucléé d'allure fibro-histiocytaire formant des plages et d'un contingent de cellules géantes multinucléées

**Figure 5:**
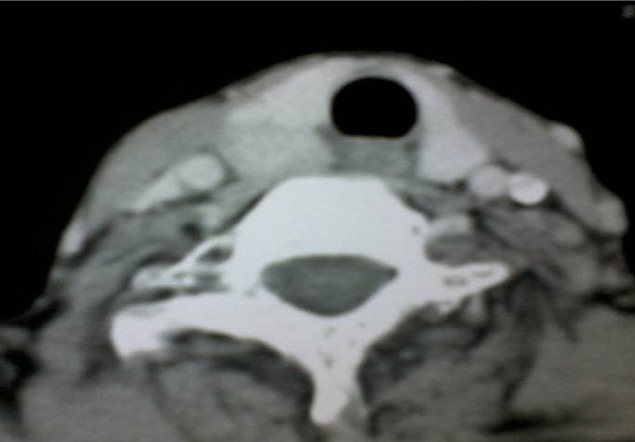
Pièce de maxillectomie

Se basant sur les données de l'examen clinique, radiologiques objectivant une tumeur agressive localement, et le résultat de l'examen anatomopathologique en faveur de la malignité, l'intervention chirurgicale a été décidée. La patiente a subi une maxillectomie médiane droite par voie transfaciale, l'exérèse de la tumeur est réalisée en monobloc emportant l'os nasal droit, le processus frontal du maxillaire, l'os lacrymal, la paroi latérale de la fosse nasale, le plancher de l'orbite dans sa partie antéro-interne, arrivant en dehors jusqu’à l'os zygomatique, et emportant en arrière la tubérosité maxillaire, en bas les 2/3 droit du palais osseux. La pièce mesure 6/5,5/4 cm, comporte 2 molaires et une incisive (Figure 4). La reconstruction a consisté en la mise en place d'un obturateur provisoire en silicone, en attendant la prothèse définitive. L'aspect histopathologique était celui d'une tumeur à cellules géantes bénigne grade I et II, compte tenu le siège inhabituel, le diagnostic différentiel se pose avec un granulome réparateur à cellule géantes et une tumeur brune dans le cadre d'une hyperparathyroïdie. Pas de signe de malignité décelable (Figure 5). Devant l'association des données: radiologiques: image ostéolytique, et histologiques: tumeur à cellule géante, le diagnostic de tumeurs brunes des maxillaires à été évoqué. Le bilan phosphocalcique a montré, une hypercalcémie à 133 mg/L et une hypophosphorémie à 17 mg/L. Les phosphatases alkalines étaient à 227 U/L (normal range 30 à 100 U/L); le bilan rénal (urée, créatininémie) était normal. La protidémie était normale. Le bilan sanguin a suggéré le diagnostic d'hyperparathyroïdie primaire initialement exprimé par une tumeur brune du maxillaire, ce qui a poussé les investigations à la recherche de l’étiologie en cause. L’échographie cervicale a permis de déceler un nodule hypoéchogène basi-cervical gauche rétro thyroïdien droit évoquant un adénome parathyroïdien.

**Figure 6:**
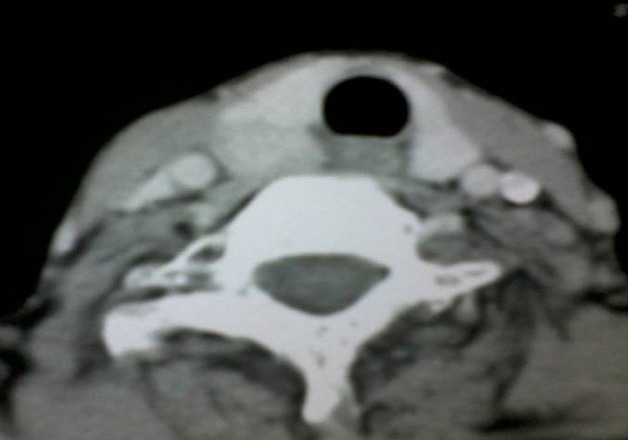
TDM cervicale en coupe axiale injectée montrant un nodule de 3,5x 2 x 1,5 cm, rehaussé de façon hétérogène, au contact des artères carotides et sub-clavières droites, et en arrière du pole inférieur du lobe thyroïdien homolatéral. Déminéralisation osseuse multi-géodique

La tomodensitométrie cervicale, a conforté le diagnostic en montrant une masse bien limitée, hétérodense, mesurant 35x 20 x15mm, en situation rétro et infra thyroïdienne droite, refoulant le lobe droit en haut et en avant en faveur d'un adénome parathyroïdien (Figure 5). Le dosage de la PTH a montré un niveau très élevé à 322 pmol/L (valeurs normales: 9-55), L'ablation de l'adénome parathyroïdien a été réalisée après 1 mois du diagnostic. La masse mesurait 3.5× 2.5 cm (Figure 6). Le rapport anatomopathologique était en faveur d'un adénome parathyroïdien.

Une scintigraphie osseuse a montré une hyperfixation diffuse de l'ensemble du squelette: de la voute crânienne plus marquée au niveau des maxillaires, des humérus, des sacro-iliaques, et du pubis. Deux mois après la parathyroïdectomie, le bilan sanguine, y compris: la calcémie et la phosphorémie, est redevenu normal. Le dosage de la parathormone s'est normalisé après quatre mois 33 pmol/L.

## Discussion

Actuellement, l'hyperparathyroïdie est de découverte fortuite dans 75 à 80% des cas à l'occasion d'un bilan sanguin montrant une hypercalcémie asymptomatique [7]. Elle peut aussi être révélée par des lithiases rénales ou des troubles cardiovasculaires [8].

Les manifestations osseuses de l’ hyperparathyroïdie: les kystes osseux, l'ostéoporose, la résorption sous périostée et les tumeurs brunes, représentent l'expression tardive de la maladie, elles sont devenues rares et surviennent dans 5 à 15% des cas [9, 10].

Par ailleurs, il est exceptionnel qu'une tumeur brune soit le premier et le seul témoin de l'hyperfonctionnement parathyroïdien [4]. L'incidence de ces lésions rapportée il y a une vingtaine d'années était de 1,5 à 1,7% dans l'hyperparathyroïdie secondaire et de 3% dans l'hyperparathyroïdie primaire [11, 12].

L’ hyperparathyroïdie est liée, dans la majorité des cas à une hyperparathyroïdie primaire. Cette dernière résulte dans plus de 80% des cas d'un adénome parathyroïdien, plus rarement d'une hyperplasie (15%) [13, 14].

L’ hyperparathyroïdie primaire touche fréquemment les patients âgés de plus de 50 ans, particulièrement chez les femmes en post ménopause. Notre patiente était en activité génitale normale, elle était âgée de 32 ans. Depuis l'introduction du dosage systématique du calcium dans les analyses biologiques de routine et le développement des techniques de dosage de la 1'84 parathyroid hormone, le diagnostic est fait fortuitement chez des patients asymptomatiques ou lors du bilan d'ostéoporose.

Il existe une prédilection pour le sexe féminin dans les hyperparathyroïdies bénignes [15]. Les tumeurs brunes peuvent affecter tout le squelette, les localisations les plus fréquentes sont le bassin, les côtes, les fémurs, la mandibule et les mains. La localisation maxillaire est extrêmement rare [16].

Cliniquement, les symptômes causés par ces lésions dépendent de la taille du processus et de sa localisation. En effet, la tumeur brune peut se présenter sous un aspect commun à d'autres tumeurs et pseudotumeurs des maxillaires à savoir une tuméfaction osseuse jugale, palatine, et/ou gingivale avec déformation et asymétrie du visage, douleur et mobilité voire chutes dentaires [7], c'est le cas chez notre patiente. Chez d'autres patients, la lésion peut être asymptomatique et le diagnostic est fait fortuitement suite à un examen radiologique systématique.

Radiologiquement, la tumeur brune se manifeste par une ostéolyse non spécifique pouvant prendre plusieurs aspects. L'aspect le plus commun est celui d'une lyse osseuse monogéodique ou multiloculaire à limites non précises entraînant une soufflure des corticales voire leur rupture, l'aspect peut alors suggérer la malignité notamment pour les lésions destructives. Le scanner révèle une masse de densité tissulaire, prenant le produit de contraste, mais qui n'envahit pas les tissus mous et aucune réaction périostée n'est remarquée [17]. Le sinus maxillaire est fréquemment comblé par la lésion qui réalise une masse intra sinusienne. Les radiographies standards du squelette sont demandées à la recherche d'autres localisations, des lithiases rénales ou une néphrocalcinose.

Les autres manifestations radiologiques de l’ hyperparathyroïdie incluent la résorption sous périostée, typiquement localisée au niveau des phalanges [18]

L’échographie et la TDM cervicales sont demandées pour détecter une lésion des glandes parathyroïdes à l'origine de l'hyperparathyroïdie.

Le scanner après injection du technetium (Tc-99m), est le meilleur moyen pour localiser la lésion au niveau des glandes parathyroïdes ou des tissus ectopiques avant la chirurgie [8]. La tumeur brune fait partie des lésions à cellules géantes des maxillaires regroupant en outre, la tumeur à cellules géantes vraie (ancienne tumeur à myéloplaxes), le granulome réparateur central à cellules géantes GRCCG, et le kyste anévrysmal [4, 7]

Dans une étude rétrospective à propos de 32 lésions osseuses à cellules géantes durant 20 ans, sept étaient localisées dans la région de la tête et du cou, quatre granulomes réparateurs à cellules géantes, trois étaient des tumeurs à cellules géantes vraies [19-26], ce qui illustre le caractère exceptionnel de la tumeur brune.

La distinction entre la tumeur brune et les autres lésions à cellules géantes est difficile, en se basant seulement sur les données de l'histologie. En effet, les tumeurs brunes se manifestent par des transformations histologiques non pathognomoniques pouvant être observé dans une tumeur à cellules géantes vraie, le granulome réparateur, kyste anévrysmal et dans une dysplasie fibreuse [20, 7].

D'où la nécessité de prendre en considération l'histoire clinique, et les résultats des examens biologiques et surtout le statut hormonal, la mise en évidence d'une perturbation du bilan phosphocalcique fait suspecter une hyperparathyroïdie, l'augmentation du taux de la parathormone permet d’établir le diagnostic [7].

Il est important de souligner que les tumeurs brunes sont des lésions non néoplasiques, sans aucun potentiel malin, comparativement avec les tumeurs à cellules géantes vraies qui ont un potentiel de transformation maligne et exposent à des métastases pulmonaires, nécessitant donc un traitement chirurgical radical. Le granulome réparateur est une autre lésion à distinguer de la tumeur brune, il s'agit d'une tumeur localisée retrouvée chez la jeune population. La pathogénie reste inconnue, cependant, certains auteurs évoquent le traumatisme comme facteur déclenchant [20].

Il est logique de considérer que le traitement de l’ hyperparathyroïdie doit être la première étape dans la prise en charge de ces patients. En effet, il existe un consensus général qui considère que la para thyroïdectomie, est le traitement de choix de l'hyperparathyroïdie primaire, cependant, les opinions se divisent quand au traitement des lésions osseuses secondaires. En effet, l’évolution des tumeurs brunes après la para thyroïdectomie est variable en fonction de leur composition [21]. La plupart des auteurs, croient que la régression spontanée de ces lésions est possible après la correction de l'hyperparathyroïdie [7], avec remplacement des lésions osseuses en un tissus osseux normal. Selon certains auteurs [6], la disparition complète de la lésion est retrouvée six mois après le traitement de l'hyperparathyroïdie. Cependant, certains [22], ont rapporté que la régénération osseuse spontanée peut durer plusieurs années avant de restaurer une morphologie normale de la face [5]. Dans les cas où les lésions sont extensives ou largement destructives, affectant la fonction de l'organe, les dommages tissulaires engendrés ne peuvent être réparé malgré l'obtention d'une normo calcémie. Dans ces situations, ou lorsque les lésions persistent après le traitement de l'hyperparathyroïdie, ou lorsqu'elles continuent à croitre malgré le contrôle hormonal, Yamazaki [2] recommande le curetage de la tumeur et son énucléation.

Pour Cicconetti [4], la première étape est l'exérèse chirurgicale de la tumeur afin de stopper la destruction osseuse, suivie d'une seconde intervention, la para thyroïdectomie, visant la suppression de la sécrétion de la parathormone.

Chez notre patiente, l'intervention chirurgicale était guidée par la biopsie préopératoire révélant une tumeur maligne, et le caractère invasif de la tumeur au scanner. La tumeur mesurait 6 cm de grand axe avec un risque imminent d'envahissement intra-orbitaire: plancher de l'orbite lysé au scanner avec infiltration de la graisse extra-conique.

## Conclusion

L'hyperparathyroïdie Primaire ou secondaire peut se manifester uniquement par la présence de lésions ostéolytiques des os de la face. Par conséquent, une lésion à cellules géantes des maxillaires doit faire rechercher systématiquement une hyperparathyroïdie par la pratique d'un bilan phosphocalcique et un dosage de la parathormone.

Le diagnostic de l'hyperparathyroïdie primaire permet d’éviter d'opérer les tumeurs brunes des maxillaires qui devraient régresser après l'exérèse de la lésion parathyroïdienne. L'observation présente a démontré la difficulté d’établir un diagnostic correct chez les patients avec un processus ostéolytique des maxillaires se présentant en histologie comme une lésion à cellule géantes, qui est crucial pour proposer un traitement approprié.
